# State of Health Prediction of Lithium-Ion Battery Based on Deep Dilated Convolution

**DOI:** 10.3390/s22239435

**Published:** 2022-12-02

**Authors:** Pengyu Fu, Liang Chu, Jihao Li, Zhiqi Guo, Jincheng Hu, Zhuoran Hou

**Affiliations:** 1 College of Automotive Engineering, Jilin University, Changchun 130022, China; 2 Department of Aeronautical and Automotive Engineering, Loughborough University, Loughborough LE11 3TU, UK

**Keywords:** State of Health (SOH), convolutional neural network (CNN), dilated convolution, one-dimensional convolution

## Abstract

A battery’s charging data include the timing information with respect to the charge. However, the existing State of Health (SOH) prediction methods rarely consider this information. This paper proposes a dilated convolution-based SOH prediction model to verify the influence of charging timing information on SOH prediction results. The model uses holes to fill in the standard convolutional kernel in order to expand the receptive field without adding parameters, thereby obtaining a wider range of charging timing information. Experimental data from six batteries of the same battery type were used to verify the model’s effectiveness under different experimental conditions. The proposed method is able to accurately predict the battery SOH value in any range of voltage input through cross-validation, and the SDE (standard deviation of the error) is at least 0.28% lower than other methods. In addition, the influence of the position and length of the range of input voltage on the model’s prediction ability is studied as well. The results of our analysis show that the proposed method is robust to different sampling positions and different sampling lengths of input data, which solves the problem of the original data being difficult to obtain due to the uncertainty of charging–discharging behaviour in actual operation.

## 1. Introduction

Compared with traditional fuel vehicles, electric vehicles use battery power to provide the motive force, effectively alleviating energy consumption and environmental pollution [1,2,3]. The lithium-ion battery is the most widely used battery type in electric vehicles due to its high energy density, considerable output power, and long life cycle [4]. However, if lithium-ion batteries cannot be appropriately used, their high energy can lead to dire consequences [5]. Therefore, existing high power batteries often use rigorous packaging technology to ensure the stability of the battery. This leads to the subsequent issue that the internal state of the battery is difficult to observe directly. In all internal states, the SOH is used to describe the degradation of lithium-ion batteries over the entire battery life cycle, which can provide a reference for battery control strategies to ensure the performance and reliability of the entire battery system [6].

According to different battery performance requirements, SOH can be expressed by various battery states [7]. Among them, the most commonly used is the battery’s energy-related health state, reflected by the capacity degradation. The SOH is greatly affected by external factors, such as temperature, charging rate, and discharge depth. When different external environmental conditions lead to different internal states, the ageing mode of the battery may be very different [8,9]. In traditional direct measurement methods [10,11] and model-driven methods [12,13,14], researchers seek to explore the relationship between external factors and internal aging mechanisms under different environmental impact factors through measurement or modelling. However, the working environment of the battery is changeable and the aging mechanism is complex, making the experimental and labour costs enormous.

In recent years, various data-driven methods have been used to predict SOH. Data-driven methods identify the aging mode of the battery from large-scale data. Furthermore, they do not need to model and analyze each working environment, making for flexible SOH prediction [15]. Generally, these methods are divided into two categories, namely, differential analysis (DA) methods [16,17,18] and machine learning methods [19]. DA methods amplify the change trend of battery features by differentiating the characteristic data of the battery and establishing a mapping relationship between the differential eigenvalue and SOH. DA methods have achieved good prediction accuracy in SOH prediction tasks, and the calculation process is relatively simple. However, the results of the DA method are susceptible to the selection of differential intervals and characteristic points. Therefore, when the DA method is used in practice, it is challenging to accurately determine them, which leads to substantial uncertainty in the prediction results, especially in online prediction.

Compared with DA methods, machine learning methods focus on mining the nonlinear relationship between battery features and SOH. According to the method of obtaining the nonlinear relationship, machine learning methods can be divided into two subcategories: algorithms based on kernel functions [20,21,22], and neural network methods [23]. Machine learning algorithms using kernel functions include support vector regression (SVR) [24], relevance vector machine (RVM) [25], and Gaussian process regression (GPR) [26]. When dealing with nonlinear mapping, mapping the data in the input space to the high-dimensional feature space is necessary. Using the kernel function, the inner product operation of high-dimensional space can be transformed into the kernel function calculation of low-dimensional input space, which ingeniously solves the problem of “dimension disaster” in the calculation of high-dimensional feature space.

An artificial neural network (ANN) is composed of multiple interconnected neurons that simulate the human neural network structure [23]. By introducing a nonlinear activation function, the artificial neuron is controlled to be in different activation or inhibition states to complete the modelling of nonlinear problems. The neural network can automatically learn the system’s input and output sample pairs, and can be used as a general mathematical model of multidimensional nonlinear functions. However, machine learning models have unavoidable limitations. Due to the limited complexity of machine learning models, they cannot analyze and obtain all features from many data. Moreover, this leads to the input of the machine learning model often manually extracting features, meaning that to a large extent the prediction result of the machine learning model does not depend on the model’s performance and instead depends more on the quality of the input features.

With the deepening of ANN research and the continuous improvement of computing capacity, deep learning methods such as deep neural networks (DNN) [27], convolutional neural networks (CNN) [19], and recurrent neural networks (RNN) [28] have become widely used to predict SOH. By increasing the number of hidden layers, a deep learning model can obtain more nonlinear relationships, combine the features of lower layers to form very abstract high-level features, and then complete complex regression tasks through simple models. The automatic feature acquisition process replaces the manual feature screening process that requires professional knowledge, and can approximate any complex nonlinear mapping with precision. However, deep learning methods often require full battery charging–discharging data. In practical applications, the charging process is relatively stable, and it is not easy to ensure that the charging–discharging range of each cycle is the same. Therefore, applying a battery health prediction method that requires complete charge and discharge data online is difficult. However, limited battery data significantly decreases the accuracy of the battery health prediction model, as the original use scenario of the existing deep learning methods is generally not battery data. Furthermore, the ability to extract the features of battery data is poor; in particular, there is difficulty in obtaining the correlations between various types of battery data.

To solve this problem, this paper develops a dilated convolution-based SOH prediction method. Dilated convolution injects holes based on standard convolution, which can obtain more comprehensive information about the charging range [29]. At the same time, one-dimensional convolution is used to replace the traditional two-dimensional convolution in order to fully obtain the correlation features between different types of battery data. The significant contributions of this work are as follows:

1. With the aim of obtaining the features of battery data that are highly correlated with charging time, we propose a deep learning model based on dilated convolution. The features of long-range information are obtained through dilated convolution, and the high-level features between different types of battery data are obtained through one-dimensional convolution. Better feature acquisition capability ensures the prediction accuracy of the model.
2. Dilated convolution is able to increase the receptive field by adjusting the number of holes without increasing the number of parameters of the convolution kernel, which avoids loss of input information in the pooling layer. After multi-layer dilated convolution, the sequence length that can be convolved achieves exponential growth, which is convenient for obtaining the timing-related features of charging data.
3. Due to the particularity of battery data, there are fewer types of battery charging data and more datapoints for each type of data. During two-dimensional convolution, the length of the category dimension decreases rapidly and the depth of two-dimensional convolution is limited, which makes it difficult to obtain high-level features between different types of battery data. One-dimensional convolution avoids the problem of dilated convolution being difficult to expand in small length dimensions.

The rest of this paper is divided into four parts. Section 2 introduces a number of related works. Section 3 introduces the battery experiment and data processing. Section 4 introduces the methodology of this paper. Section 5 presents and analyzes the experimental results. Section 6 draws the main conclusions.

## 2. Related Work

Sun et al. [16] used Regional Capacity Analysis (RCA) and Differential Voltage Analysis (DVA) to verify battery SOH evaluation. As a novel and effective indicator, the DVA terminal slope is linearly and negatively correlated with battery SOH. Zhang et al. [17] compared incremental capacity analysis (ICA) to describe the relationship between incremental battery capacity and battery SOH. The maximum peak height in the ICA curve is linearly and positively correlated with the battery SOH. Kurzweil et al. [18] reflected the slope of the flat area of charge–discharge features through differential capacity, reflecting small changes during battery aging, which was able to reflect SOC and SOH at well the low measurement frequency.

In the kernel function algorithm, Li et al. [24] extracted four health features highly related to SOH decline from the battery charging and discharging data as the input of support vector regression (SVR) and optimized the parameters of the SVR model based on the improved ant lion optimization algorithm. Wang et al. [30] proposed a prediction model of SOH based on multi-kernel RVM, obtaining the original features from the voltage curve and temperature curve of battery charge–discharge and then screening the features through the minimum redundancy maximum correlation (mRMR) algorithm. Jia et al. [26] combined the Gaussian process regression (GPR) method with probability prediction to predict short-term SOH. In this approach, indirect health indicators (IHIs) are extracted from the voltage, current, and temperature curves during battery charging and discharging and the features are selected as the input of the SOH prediction model through the grey relationship analysis method.

The deep learning methods most commonly used for SOH prediction at present are CNN and LSTM. Khumprom et al. [27] used DNN to train multiple feed-forward layers using backpropagation random gradient descent through a stack of multiple hidden layers, achieving high prediction accuracy. Chemali et al. [19] used CNN as the basic model to verify the advantages of CNN in battery SOH prediction that is not limited by basic electrochemical process knowledge. At the same time, they used data enhancement technology to generate data for CNN training, which further improved the prediction accuracy of the CNN. Bi et al. [28] used a time convolution network (TCN) based on RNN and CNN and deduced three groups of characteristic variables with obvious correlation with SOH through the battery charge–discharge data curve, obtaining high prediction accuracy.

## 3. Data

In this section, the battery experiment steps, battery data acquisition, data preprocessing methods, and experimental dataset division methods are explained in detail.

### 3.1. Battery Aging Test

The aging test in this paper is conducted on commercial 21,700 Nickel cobalt aluminium (NCA) battery by the BaSyTec battery test system. The rated voltage is 3.6 V, and the rated capacity is 4.8 Ah. The operating voltage range is 2.5 V to 4.2 V. The battery in this paper is charged to 100% SOC from 0% SOC through standard constant current–constant voltage (CC–CV) charging, then discharged to 0% SOC through CC to complete one charge–discharge cycle test.

In charging mode, the battery is charged at a current rate of $I_{c}$ until the terminal voltage reaches the preset voltage value.

The CV mode is then used to depolarize until the current drops to the cut-off current of 0.02C (where *C* is the battery’s rated capacity in ampere hours).

After being put aside for 5 min, the battery discharges to the preset lower limit at a current discharge rate of $I_{d}$.

It should be noted that all experiments were conducted at room temperature (25 °C), and the temperature remained unchanged during the experiments.

### 3.2. Acquisition of SOH Value

**Figure 1:**
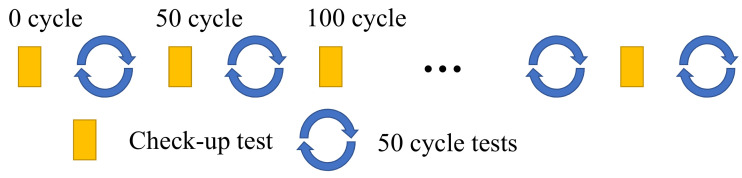
Schematic diagram for testing battery SOH in the battery aging test.

The cell capacity can be calculated via Coulomb counting by integrating the discharge current (*I*) with the time (*t*) as Q=∫Idt. The current rate of C/3 is commonly used to obtain the capacity value. The capacity check-up test was carried out with Coulomb counting every 50 cycles. The test method is shown in Figure 1. According to the capacity test results, the SOH value of each 50 cycles can be calculated by Formula (1). Then, the SOH value corresponding to each cycle can be obtained by interpolation, which is the label value corresponding to the corresponding input charge–discharge cycle data:

(1) $$\mathrm{SOH}=\frac{Q_{aged}}{Q_{fresh}}\times 100\%$$

where $Q_{fresh}$ denotes the nominal capacity at a specific charging rate when the battery is in the initial state and $Q_{aged}$ denotes the ageing capacity measured at a specific time.

### 3.3. Data Preprocessing

**Figure 2:**
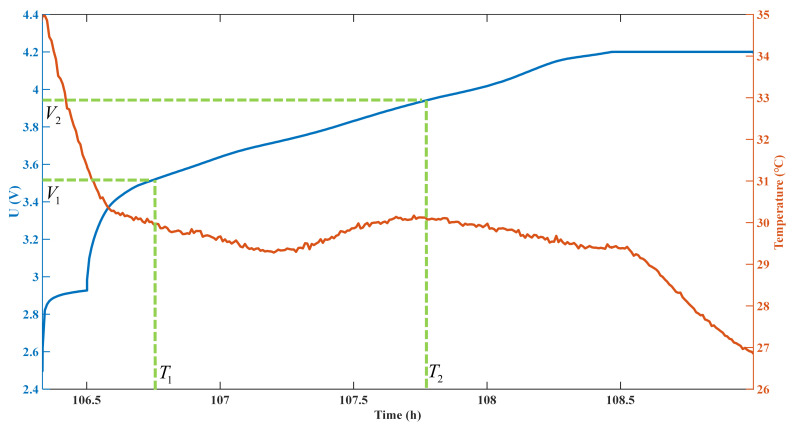
Voltage and temperature data of a cycle in the battery aging test.

Due to the battery’s aging, the charging time of the battery may be different under the same charging and discharging conditions. By contrast, in the process of constant current charging, the operating voltage range of the battery remains unchanged. Therefore, this paper uses voltage as a reference to process other types of unprocessed data. Figure 2 shows the voltage and temperature data of a randomly selected charging process.

**Figure 3:**
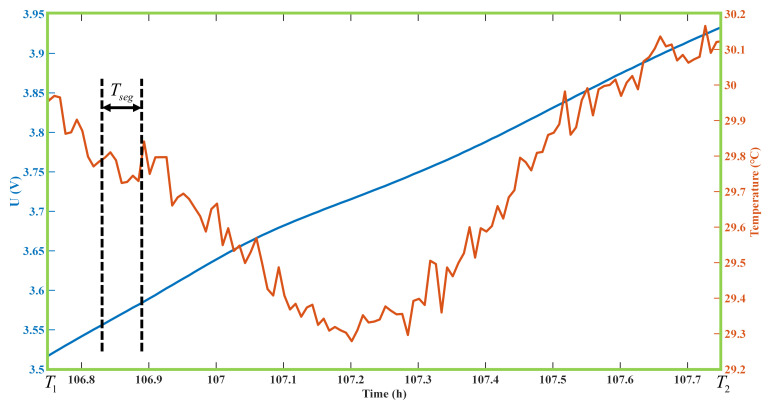
Voltage and temperature data of the corresponding time period $\left[T_{1},T_{2}\right]$ after selecting the starting voltage $V_{1}$ and ending voltage $V_{2}$.

With the starting voltage $V_{1}$ and ending voltage $V_{2}$ defined, the charging time $\left[T_{1},T_{2}\right]$ of this charging voltage is intercepted through the voltage curve. The data in this period are selected as the input data of the SOH prediction model; Figure 3 shows the data curve of the temperature at $\left[T_{1},T_{2}\right]$.

The time of the defined voltage segment is divided into *K* segments with an average length of $T_{seg}$, and $T_{seg}=\left(T_{2}-T_{1}\right)/K$. The average value in this period is taken as the data feature. The formula is as follows:

(2) $$F_{t}=\frac{1}{T_{seg}}\int_{t}^{t+T_{seg}}f\left(\tau \right)d\tau$$

where $F_{t}$ is the data feature at time *t*, $T_{seg}$ is the length of segmentation, and $f\left(\tau \right)$ is the function of battery data change with charging time. Therefore, the cell data of the defined voltage segment are processed as a feature vector $FV=\left[F_{1},\ldots ,F_{K}\right]$ with length *K*.

To improve the speed and robustness of model training, this paper uses the z-score [31] to normalize each feature vector, eliminating the impact of different battery feature dimensions on the training process. The formula is as follows:

(3) $$F_{i}^{N}=\left(F_{i}-mean\left(FV\right)\right)/std\left(FV\right)$$

(4) $$mean\left(FV\right)=\frac{1}{K}\sum_{i=1}^{K}F_{i}$$

(5) $$std\left(FV\right)=\sqrt{\frac{1}{K}\sum_{i=1}^{K}{\left(F_{i}-mean\left(FV\right)\right)}^{2}}$$

where $F_{i}^{N}$ is the normalized eigenvector element and $mean\left(FV\right)$ and $std\left(FV\right)$ are the mean and standard deviation of all elements in the eigenvector, respectively.

The same processing method is used for all unprocessed data. Finally, the processed voltage feature vector $FV_{U}$, current feature vector $FV_{I}$, and temperature feature vector $FV_{T}$ are taken as the input data.

### 3.4. Structure of the Dataset

**Figure 4:**
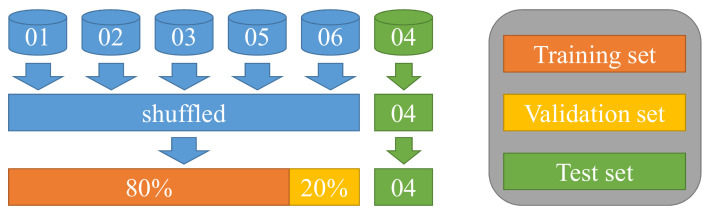
The method of dividing the experimental dataset for cross-validation.

In this paper, data from six batteries with different charging rates $I_{c}$ were used as the original data. Based on the original data, the prediction accuracy of the proposed model was cross-validated. In each test, the data on one battery in the dataset were retained for testing of the model, making for a total of six groups of tests. In addition, data from the five cells other than the test cell were randomly shuffled; 80% of the shuffled data were then selected as the training set for this test, with the remaining 20% used to form a validation set. Figure 4 shows the division process of one test dataset. The overall predicted average performance of all tests reflected the model’s final performance.

## 4. Methodologies

**Figure 5:**
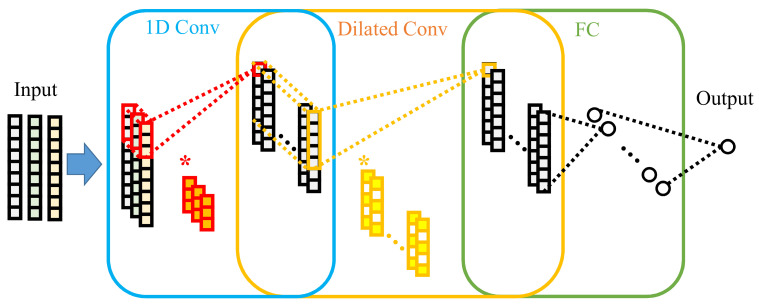
Overall structure of battery SOH prediction model based on Dilated CNN.

This paper develops an SOH prediction model based on dilated convolution, as shown in Figure 5, * is convolution operation. The basic models of this model are one-dimensional convolution and dilated convolution. In one-dimensional convolution, the relationship between the number of input features and the number of convolution layers is decoupled, which is convenient for increasing the depth of the model. The superimposition of dilated convolution layer enables the convolution layer at the top layer to obtain the timing features of an extended charging range, which improves the feature acquisition capability. The rest of this section introduces the fundamental theories of 1D convolution and dilated convolution.

### 4.1. One-Dimensional Convolution

It can be seen from the data processing approach described above that there are fewer types of battery data, while the discrete battery data feature vectors are relatively long. When traditional two-dimensional convolution is used, the length–width difference of the input feature matrix formed by the battery input feature vectors is enormous, leading to a rapid decline in the width of the feature maps. Therefore, this paper uses one-dimensional convolution to adapt the input data to the battery SOH prediction task. The formula for one-dimensional convolution is as follows:

(6) $$s\left(n\right)=f\left(n\right)*g\left(n\right)=\sum_{m=0}^{l-1}f\left(m\right)\cdot g\left(n-m\right)$$

where $s\left(n\right)$ is the convolution result, $f\left(n\right)$ is the input data, and $g\left(n\right)$ is the convolution kernel with the size of *l*.

**Figure 6:**
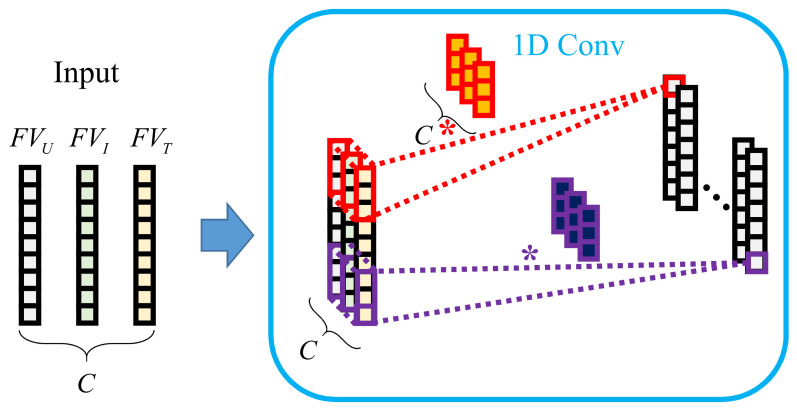
Realization of one-dimensional convolution.

As shown in Figure 6, the one-dimensional convolution of the battery SOH prediction task takes different kinds of feature vectors as the number of channels *C* of the input data, meaning that the size of the input data is C×K×1. The channels of the convolution kernel are consistent with that of the input layer, and the information on different features is obtained through multiple convolution kernels.

### 4.2. Dilated Convolution

**Figure 7:**
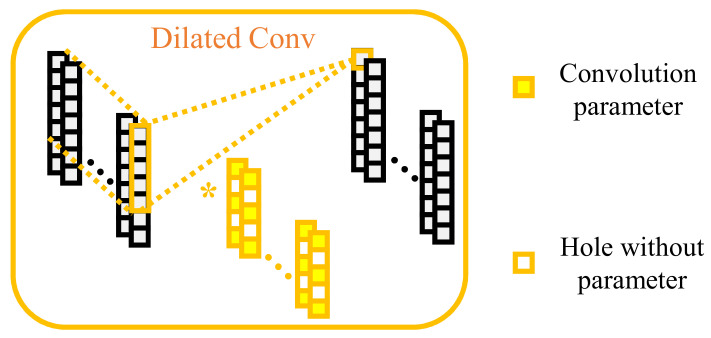
Realization of dilated convolution.

Compared with the standard convolution operation, the dilated convolution enlarges the receptive field of the convolution kernel by injecting holes without increasing the learnable parameters. Moreover, a new model hyperparameter *d* is introduced in dilated convolution, called the dilation rate, to describe the distance between the learnable parameters in the convolution kernel. Specifically, by default the standard convolution kernel’s dilation rate is one. Figure 7 shows a schematic diagram of dilated convolution. When d=2, the receptive field of the dilated convolution kernel in this layer is 5, while the learnable parameter of each channel remains 3.

**Figure 8:**
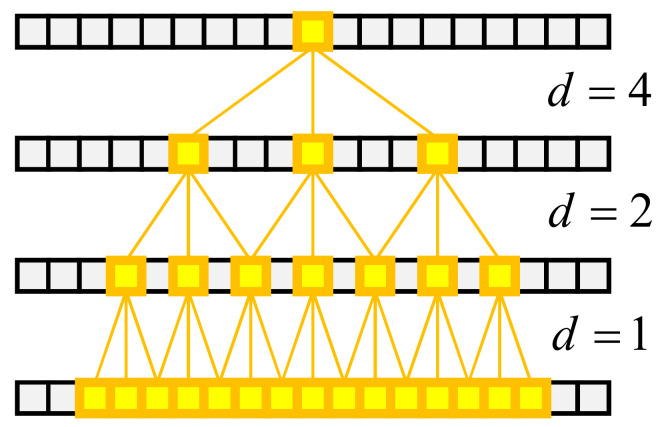
By stacking multiple layers of dilated convolution, deep neurons can obtain large receptive fields. The yellow squares are neurons that can be perceived.

After stacking multiple dilated convolutions, the receptive field corresponding to an output neuron increases exponentially, as shown in Figure 8. Through three layers of dilated convolution with different *d*, a neuron at the top layer can obtain information far away in the bottom layer. Therefore, dilated convolution can replace the role of the pooling layer in traditional convolution to expand the receptive field, effectively reducing the loss of original information.

## 5. Experiment

Based on the existing data, we designed three experiments to verify the prediction effect of the proposed model. This section introduces the evaluation indicators, implementation details, and results of three groups of experiments.

### 5.1. Evaluation Indicators

To evaluate the SOH prediction effect of various models, this paper uses the root mean square error (RMSE), correlation coefficient ($R^{2}$), and standard deviation of error (SDE) as the evaluation criteria. The formulas are as follows:

(7) $$\mathrm{RMSE}=\sqrt{\frac{1}{m}\sum_{i=1}^{m}{\left(y_{i}-{\hat{y}}_{i}\right)}^{2}}\times 100\%$$

(8) $$\mathrm{SDE}=\sqrt{\frac{1}{m}{\sum_{i=1}^{m}\left(x_{i}-\bar{x}\right)}^{2}}$$

(9) $$R^{2}=1-\frac{\sum_{i=1}^{m}{\left(y_{i}-{\hat{y}}_{i}\right)}^{2}}{\sum_{i=1}^{m}{\left(y_{i}-\bar{y}\right)}^{2}}$$

where *m* is the amount of data participating in the error calculation, $y_{i}$ is the real value of SOH, ${\hat{y}}_{i}$ is the predicted value of SOH, $x_{i}=y_{i}-{\hat{y}}_{i}$ is the error of SOH, and $\bar{x}$ is the average value of the error. Smaller values of RMSE and SDE indicate a better prediction effect, with $R^{2}\in \left[0,1\right]$ and $R^{2}$ close to 1 being the best.

### 5.2. Implementation Details

The experiments were run on an Intel Core processor with an i5-12600k CPU, NVIDIA GTX 1080 GPU, and 32 GB RAM. The Adam optimization algorithm [32] with momentum was used to reduce the generalisation error. Grid search was used, with iteration over all candidate parameters, every possibility tried, and the best parameter taken as the final result. The batch size was set to 128, the training epochs to 4000, and the learning rate to 0.001 for all model parameters. In addition, we set a dropout of 0.1 to alleviate overfitting problems in the training process. Table 1 summarizes several essential parameters in training the battery SOH prediction model.

The SOH prediction model in this paper consists of two parts: one is a three-layer one-dimensional dilated convolution, and the other is a three-layer full connection. The network configuration is shown in Table 2.

Here, the processed voltage feature vector $FV_{U}$, current feature vector $FV_{I}$, and temperature feature vector $FV_{T}$ are taken as the input data. In addition, the length of the feature vectors is K=50.

### 5.3. Baseline

To prove the advantages of the cell SOH prediction model based on dilated convolution, this paper provides the results of a two-dimensional convolution-based SOH prediction model for comparison. In addition, three existing SOH prediction machine learning methods are introduced: one based on support vector regression (SVR), one on Gaussian process regression (GPR), and the other on random forest (RF). Specifically, the same battery data preprocessing method is used to obtain the voltage feature vector $FV_{U}$, current feature vector $FV_{I}$, and temperature feature vector $FV_{T}$. When using two-dimensional convolution, the input feature vectors are side by side to form a two-dimensional input matrix with a channel of 1, and the dimension is 1×C×K. The machine learning methods connect the input feature vectors into a one-dimensional data vector with length C∗K, which is used as the model’s input to learn the nonlinear mapping relationship between the input features and SOH.

GPR: GPR is a nonparametric model that uses an a priori Gaussian process (GP) to perform regression analysis on data. In this paper, the radial basis function (RBF) was used as the kernel function, and the length of the kernel was set to 5.

RF: The random forest regression model establishes multiple unrelated decision trees by randomly extracting samples and features, obtaining the prediction results by averaging the results of all trees. The number of iterations was set to 100.

SVR: SVR is generally applied to scenes with sparse features and a small number of features by fitting values through hyperplanes. This paper used the radial basis function (RBF) as the kernel function; the regularization parameters and gamma values were set to 100 and 0.01, respectively.

CNN: All features were obtained through one layer of two-dimensional convolution. The size of the convolution kernel was set to [5,3], the step was set to [3,1], and there was no padding. Finally, the output was obtained through two fully-connected layers, with the first layer having sixteen hidden neurons.

### 5.4. Prediction of CC Charging Process

**Figure 9:**
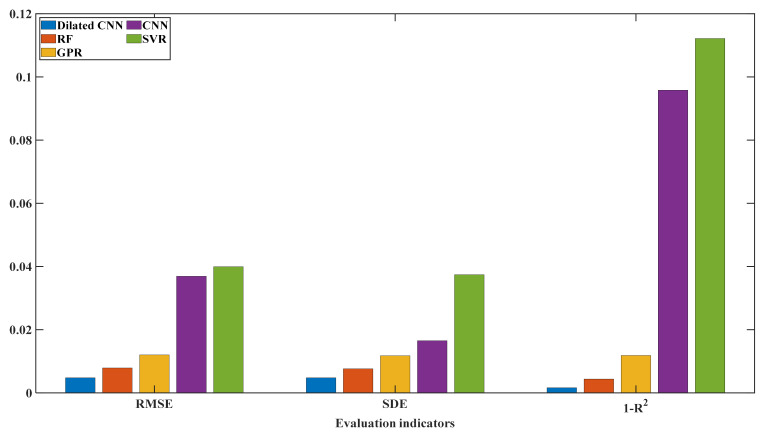
Prediction results of different baseline methods.

Except for the charging rate, the six batteries used in this paper had the same experimental conditions; thus, each battery’s voltage range for CC charging was from 2.5 V to 4.2 V. With the data on this voltage segment (2.5 V, 4.2 V) as the input data of the model, six complete independent experiments were carried out according to the cross-validation method described in Section 3.4. Finally, each model’s evaluation indicators’ average results are shown in Table 3 and Figure 9. It can be seen that the evaluation indicators of the SOH prediction result of the Dilated CNN are better than those of the other four methods, followed by the RF and GPR machine learning algorithms. The worst prediction results are with SVR and two-dimensional CNN. Except for SDE, the other evaluation indicators are not at the same level as the other three methods. These results show that the proposed method has advantages over traditional CNN in extracting battery data features after the convolution method is changed, and is able to obtain good prediction results.

**Figure 10:**
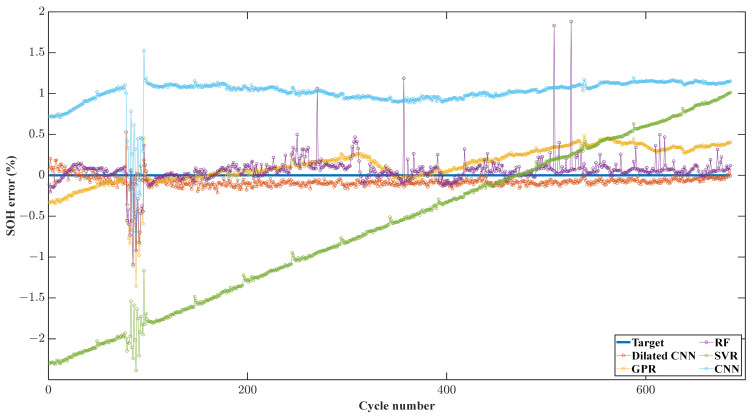
The prediction error diagram of the experiment, with cell 02 as the test set.

More specifically, Figure 10 shows the predicted SOH error diagram of each cycle of Cell 02 when Cell 02 is used as the test set. It can be seen that Dilated CNN provides the best estimate of the battery life cycle. The results wth SVR show poor performance, and it is difficult to even follow the trend of battery degradation. Although CNN predicts the trend of battery degradation, it shows apparent deviation. The RF results follow the battery’s SOH value in most cases, although it occasionally shows noticeable fluctuations. The results with GPR show obvious inaccuracies at the beginning and end of the life cycle.

### 5.5. Influence of Segment Positions on Prediction Effect

**Figure 11:**
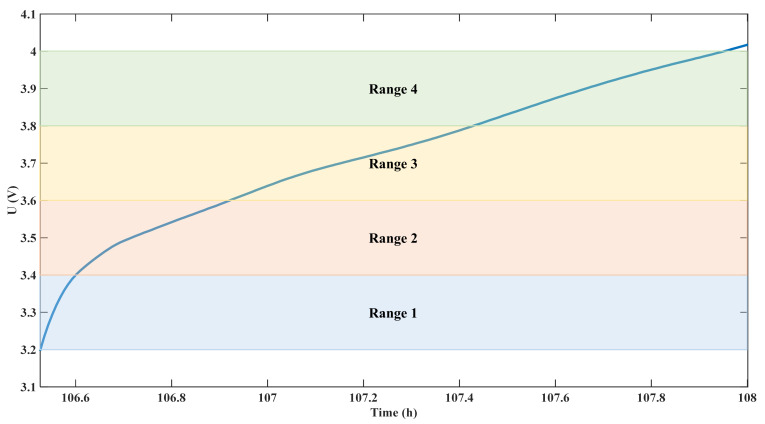
Method for dividing voltage ranges at different positions.

The voltage segment (2.5 V, 4.2 V) is the whole CC-step, corresponding to a large SOC range. In order to conform to the actual situation, (3.2 V, 4 V) in the middle is taken as the experimental segment. In order to study the influence of the position of the voltage range on the prediction results, the 3.2 V–4 V voltage range is divided into four voltage ranges on average, as shown in Figure 11.

**Figure 12:**
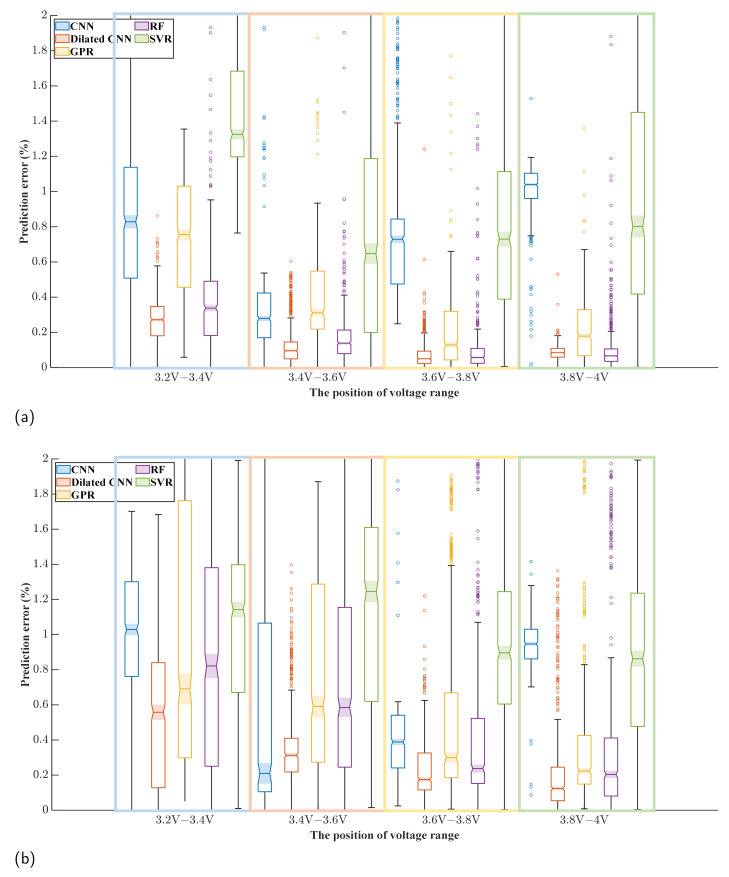
(**a**) Box chart of prediction error for different methods when using input data of voltage range at different positions with Cell 02 as the test set. (**b**) Box chart of prediction error for different methods when using input data of voltage range at different positions with Cell 04 as the test set.

Figure 12 shows a box diagram of the error of the test set with different input positions. It can be seen from Figure 12a that Dilated CNN has the best prediction effect in all input voltage ranges, with the lowest median prediction error and the most concentrated error distribution. As the position of the input voltage range grows closer to the end of the CC process, the predicted results of Dilated CNN, GPR, and RF become better. In the 3.2 V–3.4 V range, where the worst prediction effects are seen, the median of the SOH error predicted by Dilated CNN is 0.2% and 0.4% in Cells 02 and 04, respectively, which is ahead of the next-best RF method. By contrast, although the error distributions of CNN and SVR are more concentrated, the median of error increases and the prediction effect worsens. Similar conclusions can be reached based on Figure 12b, with Dilated CNN again showing the best prediction performance.

### 5.6. Influence of Segment Length on Prediction Effect

It can be seen from Section 5.5 that all methods achieve relatively good results when the voltage range is 3.6 V. Therefore, four voltage ranges with equal starting voltage and different ending voltage were selected in the constant current charging stage according to Table 4 in order to study the influence of the length of the voltage range on the prediction effect.

**Figure 13:**
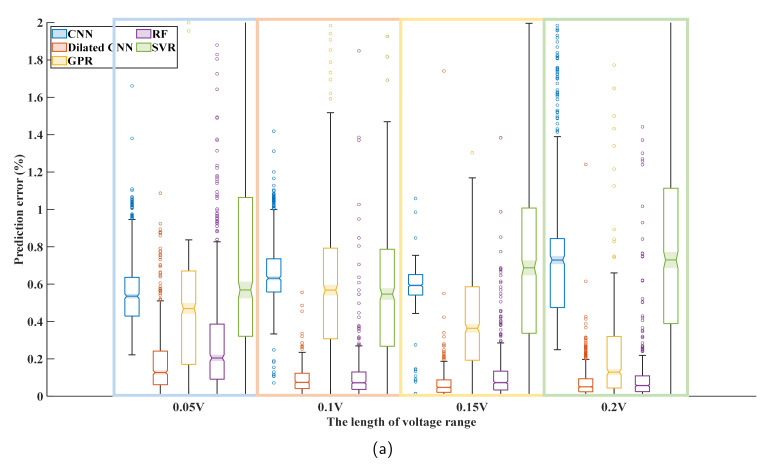

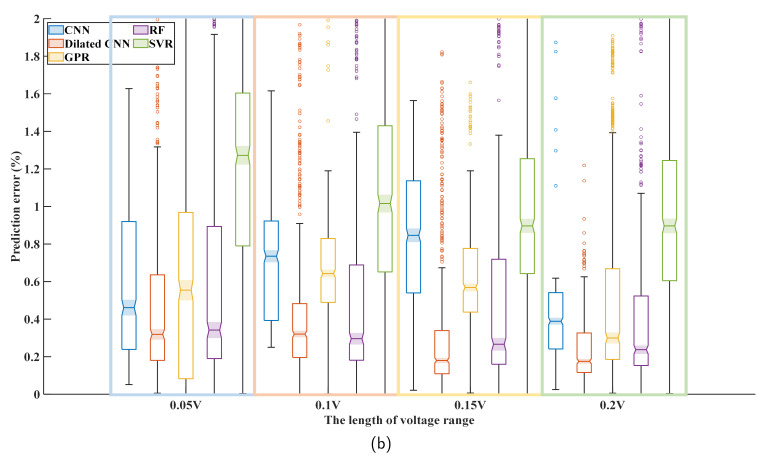
(**a**) Box chart of prediction error for different methods when using input data of voltage range at different lengths with Cell 02 as the test set. (**b**) Box chart of prediction error for different methods when using input data of voltage range at different lengths with Cell 04 as the test set.

Figure 13 shows a box diagram of the error of the test set with different input lengths. The prediction effect of dilated convolution is ultimately better than that of other methods. For most methods, increasing the length of the voltage range reduces the prediction error of SOH. However, the prediction effect of CNN and SVR methods in Cell 02 becomes worse with the increase in voltage range length. In addition, the RF method shows a tremendous difference from the different battery prediction experiments, and its robustness is poor. For Dilated CNN, the prediction error decreases obviously with the increase in the voltage range length from 0.05 V to 0.15 V. In contrast, the optimization of the prediction effect is limited when the voltage range length increases from 0.15 V to 0.2 V. It can be inferred that Dilated CNN does not require a long voltage range to ensure the prediction effect of SOH. For the remaining tests, a similar trend in the prediction error was observed, proving that the above observations are not the incidental.

## 6. Conclusions

In this study, a prediction model for lithium-ion battery SOH based on dilated convolution is proposed. In this model, a dilated convolution layer was used to replace the standard convolution layer, which can increase the receptive field of the convolution kernel without using the pooling layer. This processing method avoids the loss of a large amount of information from the original data during pooling. At the same time, compared with standard convolution kernel, a diluted convolution kernel has injected holes, meaning that the parameters of the convolution kernel are not increased and the computational efficiency is not affected. In addition, based on the significant difference between the length and width of the battery input matrix, this paper introduces one-dimensional convolution and treated different types of battery eigenvectors as the input features of different channels, which is conducive to increasing the depth of the network. In addition, multi-layer dilated convolution realizes an extensive receptive range which is able to fully extract the time feature information of the battery charging process.

According to the prediction results, when using data for any range in the CC stage as input, the proposed dilated CNN model shows superior performance to many existing methods, including two-dimensional CNN, RF, GPR, and SVR. Further research shows that the accuracy of SOH prediction is improved by extending the charging range length and selecting the back voltage range data as the model’s input. However, the method proposed in this paper achieves good prediction results when the data of the smaller charging range length and earlier voltage range are used as the model’s input. The depth learning model based on dilated convolution proposed in this paper is robust to different sampling positions and different sampling lengths of input data, which solves the problem of the original data being difficult to obtain due to the uncertainty of charging–discharging behaviour in actual operation.

However, such robustness remains insufficient to support online estimation of SOH. Our future goal is to explore how to improve prediction accuracy when battery data collected from actual operation of a vehicle are used as the input, as well as whether the task of SOH prediction of battery data based on laboratory environment testing can be migrated to the online SOH prediction task. These outstanding issues prompt us to further improve our proposed deep learning method.

## Figures and Tables

**Table 1:** List of parameter values used in Dilated CNN training.

Parameter	Value
Batch size	128
learning rate	0.001
Dropout	0.1
Number of epochs	4000

**Table 2:** Configuration of Dilated CNN.

Layer	Size of Kernel	Number of Kernel	Stride	Padding	Dilated Rate	Number of Neurons
Conv.1	3	12	1	0	1	576
Conv.2	3	72	1	0	2	3168
Conv.3	3	192	1	0	4	7296
FC.1	-	-	-	-	-	256
FC.2	-	-	-	-	-	16
FC.3	-	-	-	-	-	1

**Table 3:** Prediction errors of different baseline methods.

Method	RMSE	SDE	$1-R^{2}$
Dilated CNN	0.0048	0.0048	0.0016
RF	0.0079	0.0076	0.0044
GPR	0.0120	0.0118	0.0118
CNN	0.0369	0.0165	0.0958
SVR	0.0399	0.0374	0.1121

**Table 4:** Test results for Cell 2 with transfer learning applied.

Trial	Starting Voltage (V)	Ending Voltage (V)
1	3.6	3.65
2	3.6	3.7
3	3.6	3.75
4	3.6	3.65
